# Ritonavir-Mediated Induction of Apoptosis in Pancreatic Cancer Occurs via the RB/E2F-1 and AKT Pathways

**DOI:** 10.3390/ph7010046

**Published:** 2014-01-09

**Authors:** Ramesh B. Batchu, Oksana V. Gruzdyn, Christopher S. Bryant, Aamer M. Qazi, Sanjeev Kumar, Sreedhar Chamala, Shu T. Kung, Ramana S. Sanka, Udaya S. Puttagunta, Donald W. Weaver, Scott A. Gruber

**Affiliations:** 1 Laboratory of Surgical Oncology & Developmental Therapeutics, Department of Surgery, Wayne State University, Detroit, MI 48201, USA; E-Mails: ovgruzdy@med.wayne.edu (O.V.G.); sreedharchamala94@yahoo.co.in (S.C.); skung@med.wayne.edu (S.T.K.); dweaver@med.wayne.edu (D.W.W.); scott.gruber@va.gov (S.A.G.); 2 John D. Dingell VA Medical Center, Lab 4242, 4646 John R Street, Detroit, MI 48201, USA; 3 NEA Baptist Clinic, Jonesboro, AR 72401, USA; E-Mail: Chris.Bryant@NEABC.com; 4 Ontario Institute for Cancer Research, Toronto, ON M5G 0A3, Canada; E-Mail: aamer.qazi@oicr.on.ca; 5 Baptist Memorial Medical Group, Memphis, TN 38120, USA; E-Mail: Sanjeev.kuman@bmg.md; 6 Virocan Therapeutics R&D division, Yashaswi Hospital, Guntur 522007, India; E-Mail: sramaraoson@gmail.com; 7 Acharya Nagarjuna University, Nagarjuna Nagar, Guntur 522510, India, E-Mail: pudayasri@gmail.com

**Keywords:** ritonavir, pancreatic adenocarcinoma, AKT, retinoblastoma, 2F-1

## Abstract

Recent observations suggest a lower incidence of malignancies in patients infected with HIV during treatment with Highly Active Anti-Retroviral Therapy (HAART) utilizing protease inhibitors. We investigated the effects of ritonavir, a FDA approved HIV protease inhibitor, on proliferation of pancreatic ductal adeno-carcinoma (PDAC) cell lines. Human PDAC cell lines BxPC-3, MIA PaCa-2, and PANC-1 were propagated under standard conditions and treated with serial dilutions of ritonavir. Ritonavir inhibited cell growth in a dose-dependent manner as well as activated the intrinsic apoptotic pathway in human pancreatic ductal adenocarcinoma (PDAC) cell lines. We observed down-modulation of cell-cycle promoting and up-regulation of cell-cycle inhibitory genes; enhanced interaction of retinoblastoma protein (RB) with E2F-1 transcription factor; inhibition of phosphorylation of RB, resulting in sequestration of E2F-1 and subsequent down-regulation of S phase genes; decreased interaction of E2F-1 with its consensus binding sites; inhibition of cell motility and invasiveness; and inhibition of the AKT pathway. Our results demonstrate a potential use of ritonavir as part of combination chemotherapy for PDAC. Since ritonavir is FDA approved for HIV, drug repositioning for PDAC would limit the costs and reduce risks.

## 1. Introduction

Pancreatic ductal adenocarcinoma (PDAC) is currently the fourth leading cause of cancer death, and more than 80% of patients present with distant metastases at the time of diagnosis, thereby precluding surgical resection. Systemic chemotherapy still relies on only a few drugs and has not significantly increased overall patient survival [1], underscoring the need for development of novel therapies [2].

The advent of highly-active anti-retroviral therapy (HAART) based on protease inhibitors has greatly improved the treatment of HIV so that it is now a manageable disease [3]. Recent observations point to a decreasing incidence of some cancers in patients on protease inhibitors [4], with our previous work demonstrating inhibition of cell growth and induction of apoptosis in ovarian cancer [5]. Along these lines, phase I clinical trials with nelfinavir showed partial remission with acceptable toxicity when used along with radiation and chemotherapy in patients with locally-advanced pancreatic cancer [6].

Non-phosphorylated retinoblastoma protein (RB) is a well-characterized tumor suppressor which inhibits E2F-1 transcription factor necessary for cell-cycle progression from G0/G1 to S phase [7]. Since it has been shown that the protease inhibitor ritonavir also blocks cell-cycle progression at these points [8], we hypothesized that it may act via RB by inhibiting its phosphorylation and thus preventing its deactivation.

The AKT pathway is another important regulator of cell proliferation and survival which is activated in pancreatic cancer [9]. Since inhibition of this pathway has been observed with various protease inhibitors [10,11], we also hypothesized that RNA*i*-mediated inhibition, an innate gene-silencing mechanism, along with ritonavir treatment, may be an effective combination therapy in promoting tumor regression of pancreatic cancer.

Therefore, the objective of the current study is to assess the anti-neoplastic impact of ritonavir on pancreatic cancer with regard to its specific effects on the AKT pathway and RB. We provide evidence here for the first time that ritonavir-induced signaling pathways at the level of the cell membrane result in nuclear events that block cell-cycle progression via RB protection.

## 2. Experimental

Reagents and antibodies: Ritonavir was obtained from Sequoia Research Products Limited (Pang Bourne, UK) and dissolved in DMSO. Cell viability was assayed in 96-well plates utilizing the Cell Counting Kit-8 (CCK-8; Dojindo, Gaithersburg, MD, USA). RB and E2F-1 antibodies were purchased from Millipore (Danvers, MA, USA). Cyclins, cyclin-dependent kinases (CDKs), CDK inhibitors, Bcl-2, poly (ADP-ribose) polymerase (PARP) and β actin antibodies were purchased from Santa Cruz Biotechnology (Santa Cruz, CA, USA). Antibodies against phospho-AKT and caspases, as well as SignalSilence AKT siRNA inhibition kits, were purchased from Cell Signaling Technology (Beverly, MA, USA).

Cell lines and culture: Human pancreatic tumor cell lines BxPC-3, MIA PaCa-2, and PANC-1 American Type Culture Collection (Manassas, VA, USA) were grown in sub-confluent monolayer cultures in DMEM medium containing 10% FBS, supplemented with 2 mM glutamine, 100 U/mL penicillin, and 100 µg/mL streptomycin. Cells were cultured in a humidified atmosphere of 95% air and 5% CO_2_ at 37 °C.

Cytotoxicity assays: BxPC-3, MIA PaCa-2, and PANC-1 cells were treated with serial dilutions of ritonavir ranging from 5–30 µM dissolved in DMSO. In addition, PANC-1 cells were treated with gemcitabine 0.5 µM with or without ritonavir 20 µM. Normal human fibroblasts were purchased as a cell line from ATCC (CRL-2522) and propagated in DMEM medium. The same serial dilutions of ritonavir dissolved in DMSO were added as described for the pancreatic cell lines. Standard prototype growth curves and number of viable cells were determined for each cell line (treated and control groups) in triplicate experiments using CCK-8 according to manufacturer’s instructions and absorbance was read at 450 nm in a plate reader (FluoStar Optima, BMG Labtech, Cary, NC, USA). Growth curves were plotted over 72 h as a percentage of the value of DMSO-treated controls minus the value of untreated cells on day 0. IC50 values were calculated by entering the raw data into the “Sigmaplot” software program.

Analysis of apoptosis: For fluorescent microscopic image analysis of the apoptotic cell fraction, ritonavir-treated and control cells (1 × 10^6^/mL) were mixed with annexin V-biotin and medium-binding reagent and incubated in the dark for 15 min at room temperature. Cells were then centrifuged and medium was replaced with 1× Binding Buffer containing FITC-streptavidin. Propidium iodide was added to discriminate early apoptotic from late apoptotic or necrotic cells. A portion of cell suspension (50 µL) was placed on a glass slide with cover slip and viewed immediately using a fluorescence microscope (Zeiss, AXio CamMRm Observer. A1, One Zeiss Drive, Thornwood, NY, USA) equipped with FITC and propidium iodide.

Western blot and co-immunoprecipitation assays: Cells were trypsinized, rinsed twice in PBS, and the pellet suspended in CelLytic MT cell lysis reagent (Sigma, St. Louis, MO, USA) containing a protease inhibitor cocktail tablet (Complete, Roche Applied Science, Mannheim, Germany). After a 30 min incubation, the pellet was collected by centrifugation at 4 °C for 15 min at 12,000 *g*. Blots and co-immunoprecipitations were conducted as previously described [12]. Ritonavir-treated samples were immunoprecipitated with anti-E2F-1 polyclonal antibodies, and the complex was probed with RB monoclonal antibodies.

Gene expression profiling: Briefly, PANC-1 cells, either untreated or treated with ritonavir 15 µM for 48 h, were harvested and total RNA was isolated utilizing an RNeasy kit (Qiagen Inc., Valencia, CA, USA) as described by the manufacturer. Total RNA was sent to MOgene Company (MOgene, LC, St. Louis, MO, USA) for analysis.

Electromobility shift assay (EMSA): Nuclear extracts were prepared using a nuclear and cytoplasmic extraction kit from G-Biosciences (St. Louis, MO, USA). EMSA assays were performed using the non-radioactive EMSA kit according to the manufacturer’s protocol (Light-Shift Chemiluminescent EMSA kit, Thermo Scientific, Rockford, IL, USA). The samples were run on 6% Tris-borate-EDTA gels and subsequently blotted onto nylon membranes. Biotinylated E2F-1 gel shift oligonucleotides (5′-ATT TAA GTT TCG CGC CCT TTC TCA A-3′, Santa Cruz Biotechnology, Dallas, TX, USA) were used for probing, followed by streptavidin detection.

E2F-1 knockdown PANC-1 cell line generation with Lentiviral E2F-1 shRNA: Lentiviral shRNA for E2F-1 was obtained from Sigma-Aldrich and used to infect the PANC-1 cell line. Twenty-four h post-infection, 2.5 mg/mL of puromycin was added to select for infected cells. Infection efficiency was approximately 95%. Two weeks post-selection, the resulting clones were expanded and cells were harvested.

*In vitro* cell invasion/migration and wound-healing assays: Cell migration was determined using a modified Boyden chamber and wound healing assays were conducted using the cell-scratch method, both as previously described [13].

Transfection of siRNA: SignalSilence AKT siRNA inhibition kit (Cell Signaling Technology) that specifically inhibits the expression of both AKT1 and AKT2 was used for this experiment. Briefly, PANC-1 cells were transfected with 100 nM siRNA of AKT. Cells were harvested after 48 h and analyzed for expression of AKT, and Bcl-2.

## 3. Results

### 3.1. Ritonavir Enhances Cell Death in a Dose-Dependent Fashion in Human Pancreatic Cancer Cell Lines and Augments Gemcitabine Effect

**Figure 1:**
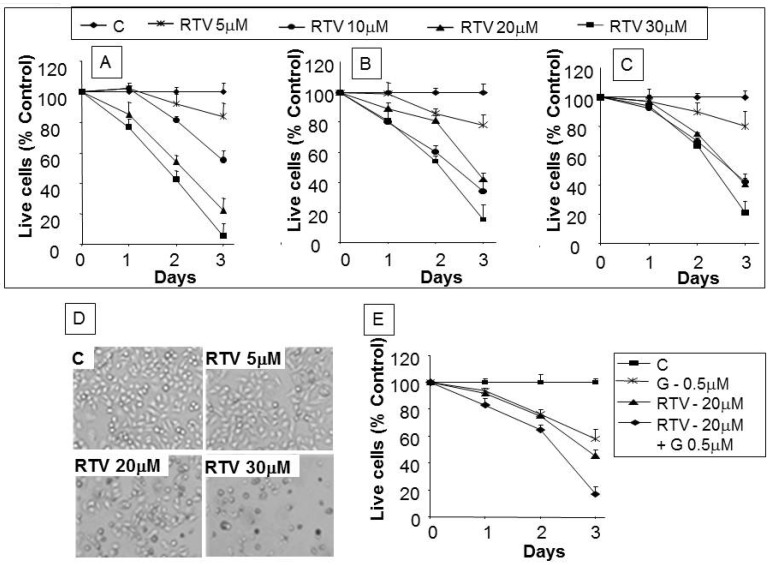
Effect of ritonavir on the growth of pancreatic cancer cell lines with enhanced killing of PANC-1 cells in combination with gemcitabine: Cells were cultured for indicated time intervals with various concentrations of ritonavir. (**A**), (**B**) and (**C**) are BxPC-3, MIA PaCa-2, and PANC-1 cell lines, respectively. Data is presented as percentage of control DMSO-treated cells and represents the mean ± SD of triplicate cultures; (**D**) Phase contrast images taken at 100× magnification 48 h after ritonavir treatment of PANC-1 cells at indicated concentrations; (**E**) Gemcitabine-mediated cytotoxicity of PANC-1 cells with or without ritonavir. C: control; RTV: ritonavir; G: gemcitabine.

Exposure to 5–30 µM ritonavir for 72 h resulted in dose-dependent inhibition of cell proliferation and cell death in all three cell lines tested (Figure 1A–C) with negligible effects on normal human fibroblasts (data not shown). At higher drug concentrations, BxPC-3 (Figure 1A) and MIA PaCa-2 (Figure 1B) demonstrated significantly greater cell death within 24 h when compared with PANC-1 (Figure 1C). IC50 over the three-day period was 7.5 µM for BxPC-3, 8.2 µM for MIA PaCa-2, and 15.5 µM for PANC-1. PANC-1 cell death as observed by phase contract microscopy at 48 h increased as a function of ritonavir dose (Figure 1D). Since gemcitabine is considered first-line chemotherapy for pancreatic cancer, we further evaluated if ritonavir increases its potency in killing pancreatic cancer cells. As shown in Figure 1E, 0.5 µM gemcitabine or 20 µM ritonavir alone produced 40.4% and 55.5% cell death respectively; however, simultaneous treatment resulted in 81.5% cell death.

### 3.2. Analysis of Apoptotic Cells with Ritonavir Treatment

**Figure 2:**
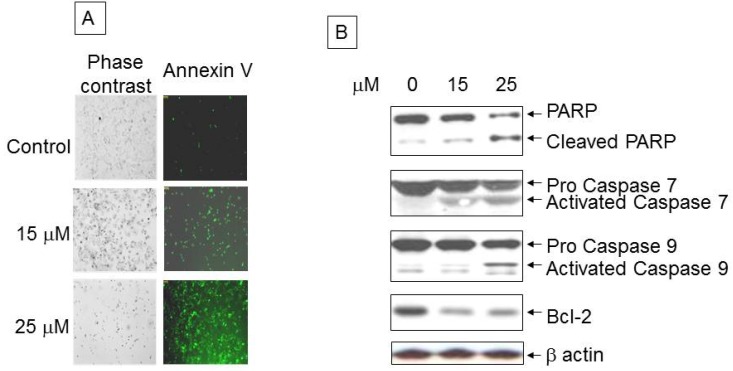
Analysis of apoptotic cells with ritonavir treatment: (**A**) Control and 24 h ritonavir-treated PANC-1 apoptotic cells within the same microscopic field were viewed and photographed by phase contrast microscopy and green fluorescence for Annexin V-Biotin-FITC staining. Using the FITC filter, early apoptotic cells appear bright green; (**B**) Western blot analysis of PARP, caspases 7 and 9, and Bcl-2 with exposure to increasing doses of ritonavir.

Immunofluorescence staining PANC-1 cells demonstrated over ~25% apoptosis following exposure to 15 µM ritonavir that increased to over 60% with 25 µM ritonavir, whereas untreated cells were less than 5% annexin V positive (Figure 2A). As shown in Figure 2B, western blot analysis revealed the activation (cleavage) of PARP as well as caspases 7 and 9 from their respective precursors. We observed decreased expression of the anti-apoptotic protein Bcl-2 with ritonavir treatment when compared with control.

### 3.3. Ritonavir-Mediated Perturbations in the Expression of Cell Cycle Regulatory Genes

**Figure 3:**
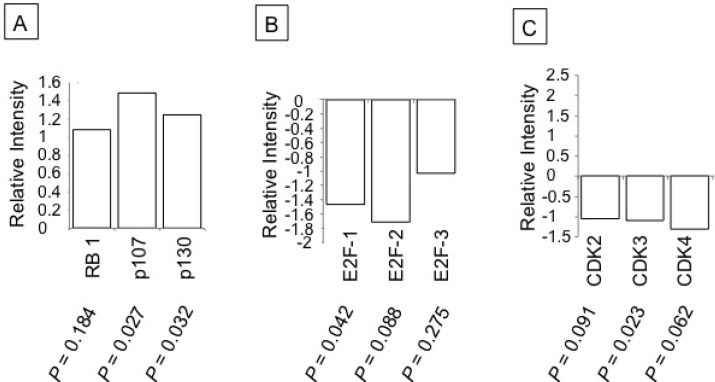
Effect of ritonavir on cell cycle genes of PANC-1 cells: PANC-1 cells treated with 25 µM ritonavir for 48 h were harvested and total RNA was isolated to generate cRNA. The cRNA was then hybridized to Whole Human Genome (G4112A) arrays according to the manufacturer’s protocol. (**A**–**C**) Gene expression of indicated cell cycle regulatory proteins. Relative Intensity: Ratio of expression of mRNA transcripts in ritonavir-treated PANC-1 cells *versus* the mock (DMSO)-treated PANC-1 cells.

Gene expression analysis of mRNA levels of RB and its related tumor suppressor proteins p107 and p130 revealed increased expression with ritonavir treatment (Figure 3A). Further, we analyzed expression levels of three members of the E2F family of proteins which interact with RB, E2F-1, -2, and -3, and observed a reduction in their expression levels (Figure 3B). Cyclins and CDKs exhibit distinct expression patterns which contribute to the temporal coordination of each event in cell cycle progression. Ritonavir treatment resulted in decreased expression of G1 phase CDKs indicating the inhibition of cell cycle progression (Figure 3C).

### 3.4. Inhibition of Cell Cycle Machinery Involved in the Phosphorylation of RB and Protection of the RB-E2F-1 Complex by Ritonavir

**Figure 4:**
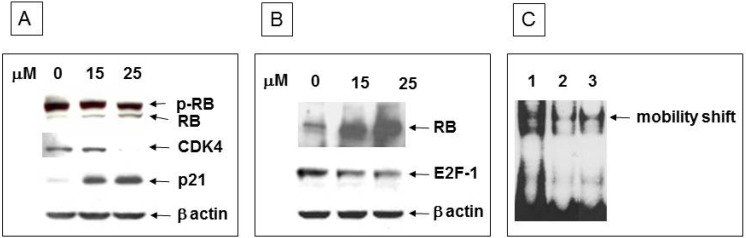
Protein expression analysis of RB-E2F-1 interaction and EMSA in PANC-1 cells: (**A**) 10 µg protein extracts of control and ritonavir-treated cells were resolved by SDS-PAGE, transferred to a nitrocellulose membrane, and probed with RB, CDK4, and p21 as indicated. β actin was used as a loading control. p-RB: hyper-phosphorylated retinoblastoma protein; (**B**) Upper panel: Indicated concentrations of ritonavir-treated cell lysates were immunoprecipitated with E2F-1 polyclonal antibody and probed with RB monoclonal antibody. Middle panel: Western blot analysis for E2F-1 in cells treated with indicated ritonavir concentrations. Bottom panel: control β actin levels; (**C**) EMSA with E2F-1 consensus binding site. Lane 1: Control nuclear extract; Lane 2: Nuclear extracts of lentiviral E2F-1 knockdown PANC-1 cell line; 3: Nuclear extracts of 25 µM ritonavir-treated cells.

To determine whether the observed growth inhibition is due to activation of RB, we analyzed the phosphorylation status of RB in response to ritonavir treatment for 24 h. We observed a >7-fold increase in the levels of non-phosphorylated RB (Figure 4A). Control of RB activity at the G0/G1 phase of the cell cycle is mainly due to the concerted action of the G1 phase cyclin CDK4 and its counterpart CKI p21. As expected, we observed lower levels of CDK4 expression in concert with significantly higher levels of p21 with increasing ritonavir exposure (Figure 4A). Non-phosphorylated RB interacts with E2F-1 and thus decreases free E2F-1 levels that are necessary for cell cycle progression. Ritonavir treatment resulted in enhanced interaction of RB and E2F-1 as evidenced by an over 10-fold increase in the levels of RB within E2F-1 immunoprecipitates and a concomitant decrease in the levels of E2F-1 protein (Figure 4B). To confirm that the lower levels of free E2F-1 are reflected in decreased binding to its promoters of cellular S phase genes, we performed an electrophoretic mobility shift assay with ritonavir-treated *versus* control PANC-1 nuclear extracts. As expected, we observed a decreased binding of E2F-1 to its consensus binding site, both in the case of ritonavir-treated as well as with RNA*i*-mediated E2F-1 knockdown cell lines (Figure 4C).

### 3.5. Ritonavir Inhibits Cell Motility and Invasiveness

**Figure 5:**
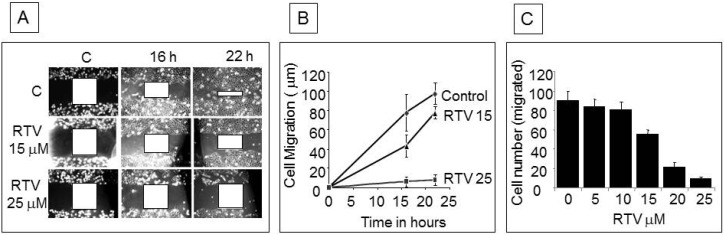
Wound healing and cell migration assays in PANC-1 cells: (**A**) Cell motility in wound healing assay. A uniform scratch was made in an 80% confluent monolayer culture and the extent of closure was monitored under phase-contrast microscopy and photographed. Representative images of two independent experiments done in duplicate are shown; (**B**) Extent of cell migration in wound healing experiment was measured at two indicated concentrations of ritonavir and plotted over time; (**C**) Chemotactic migration of cells through Boyden chamber membrane. Logarithmically growing cells were trypsinized and seeded in Boyden chambers and treated with various concentrations of ritonavir. Cells migrating through the membrane were stained and counted under the microscope. Results of three independent experiments were plotted.

Cell motility following wound generation was greater in control cells than in ritonavir-treated cells at 16 and 22 h (Figure 5A), with significant down-modulation of wound healing by 15 µM and almost complete inhibition by 25 µM ritonavir (Figure 5B). Boyden chamber experiments showed a progressive decrease in cell migration through the matrigel membrane with increasing concentrations of ritonavir (Figure 5C).

### 3.6. Ritonavir Inhibits AKT Pathway in PANC-1 Cells

**Figure 6:**
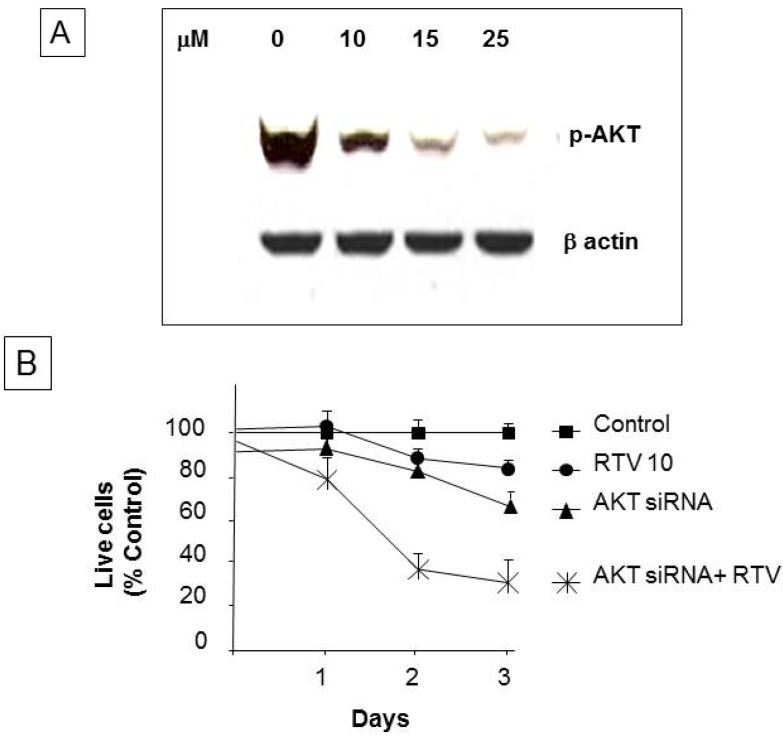
Western blot analysis for phospho-AKT and cell proliferation of AKT siRNA-treated PANC-1 cells: (**A**) 10 µg protein extracts of control and ritonavir-treated cells at the indicated concentrations were resolved by SDS-PAGE and probed with phospho-AKT antibodies. β actin was used as a loading control; (**B**) Growth inhibition by ritonavir and AKT siRNA. Cell growth is expressed as a percentage of control DMSO-treated cells and represents the mean of triplicate cultures.

We observed a dose-dependent decrease in phosphorylation of AKT following ritonavir treatment (Figure 6A). Ritonavir 10 µM and AKT 100 nM siRNA transduction each individually inhibited cell proliferation by approximately 15%–20% at 72 h, but this inhibition was dramatically increased to 60% when the two were combined (Figure 6B). Finally, we observed an even further decrease of cell growth with concomitant AKT siRNA treatment.

## 4. Discussion

**Figure 7:**
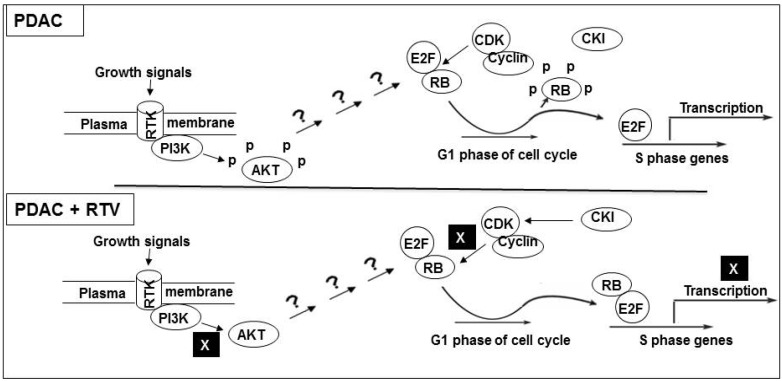
Schematic representation of signaling pathways from cell surface to nucleus modulated by ritonavir in PDAC: **Upper panel**: In the absence of ritonavir (RTV), growth signals from receptor tyrosine kinase (RTK) are conveyed to AKT via phosphotidyl inositol 3 kinase (PI3K). RB is inactivated by phosphorylation of cyclin-CDK complexes, resulting in the activation of E2F-1 transcription. E2F-1 target genes encode proteins involved in expression of S phase genes; **Lower panel**: In the presence of ritonavir, growth signals are inhibited by de-phosphorylation (activation) of RB, resulting in complex formation with E2F-1. This process inhibits transcription of S phase genes in two ways, passively via sequestration of E2F-1 and actively via the RB-E2F-1 complex.

The present study demonstrates cell cycle arrest, induction of apoptosis, and inhibition of cell migration in pancreatic cancer cell lines by ritonavir, a protease inhibitor that has been in use for over a decade in the treatment of HIV patients. Although several mechanisms are well characterized for signal transduction from AKT on the plasma membrane [14] to transcriptional activation of cell cycle control genes in the nucleus [15], the specific pathways modulated by ritonavir are not yet understood. In a recent study, ritonavir induced caspase-dependent apoptosis and suppressed NF-κB activity by inhibiting IκB phosphorylation of primary effusion lymphoma cells [16]. We provide evidence herein that in the presence of ritonavir, phosphorylation of both AKT and RB is inhibited. As a result, the ability of E2F-1, sequestered by its binding to RB, to promote transcription of necessary S phase genes is impaired (Figure 7). Since ritonavir has been shown to augment the effect of other chemotherapeutic drugs such as docetaxel [17], we conducted combination studies of ritonavir and gemcitabine, the latter considered first-line therapy for PDAC. We demonstrated an additive effect on cell death in PANC-1 cells, suggesting the benefits of ritonavir as part of combination therapy. Of note, there is a beneficial effect of combining 7-allylamino-17-demethoxygeldanamycin and ritonavir as a novel therapeutic target by inhibiting the expression of heat shock factor-1 in the treatment of renal cancer [18].

We next assessed the effect of ritonavir on the activity of enzymes involved in the apoptotic pathway. We observed activation of caspase 9, an upstream event in the intrinsic apoptotic pathway, followed by activation of caspase 7, which in turn leads to breakdown of PARP. Although untreated PANC-1 cells showed basal levels of PARP cleavage, we demonstrated a significant, dose-dependent increase in the cleavage of PARP with ritonavir exposure, along with activation of caspases and inhibition of anti-apoptotic Bcl-2.

Gene expression profiling of PANC-1 cells indicated that ritonavir could beneficially affect critical steps in cell cycle progression. Indeed, we demonstrated down-regulation of tumor-promoting E2F transcription factors and CDKs, along with up-regulation of tumor-suppressing RB proteins. More specifically, in Western blot and immunoprecipitation analyses, we found a dose-dependent (1) increase in non-phosphorylated, active RB; (2) decrease in CDK4; and (3) increase in p21^waf1/cip1^ with ritonavir exposure. These all contribute to the formation of RB-E2F-1 complexes, thereby sequestrating E2F-1, preventing subsequent activation of S phase genes, and halting cell cycle progression.

Tumor metastasis is a multistep process involving basal membrane matrix degradation, detachment of proliferating tumor cells, and transport of these cells in the blood or lymph, resulting in invasion into other tissues [19]. Our findings of decreased cell migration by the wound healing assay and inhibition of cell invasion by the Boyden chamber assay are consistent with the earlier observation that ritonavir inhibited the expression of endothelial cell adhesion molecules [20].

Our results showing ritonavir-mediated inhibition of AKT activity and decreased cell proliferation have relevance in light of earlier observations correlating gemcitabine-induced drug resistance with activation of the AKT pathway in pancreatic cancer cells [9]. We found that reduction in the expression of p-AKT by Western blot analysis correlated with decreased cell proliferation and migration. Along these lines, Yamamoto *et al.* [21] showed that low p-AKT levels were a good prognostic indicator for overall survival in 65 patients who underwent surgery for pancreatic cancer. Thus, ritonavir-induced phosphorylation (inactivation) of AKT may represent a potential therapeutic approach to management of PDAC.

Ritonavir plasma levels in HIV patients normally range from 15–45 µM, providing therapeutic efficacy with negligible side effects [22]. Of note, we observed growth inhibitory effects of ritonavir in the range of 5–20 µM, suggesting that these beneficial effects could be achieved in patients with PDAC at clinically relevant, nontoxic doses. The relatively low toxicity of these drugs, taken together with the large body of data available regarding their pharmacokinetics, tissue distribution, and safety, would allow for the rapid clinical evaluation of ritonavir alone or in combination with other chemotherapeutic agents. Indeed, recent phase I clinical trials with nelfinavir in pancreatic cancer have shown promising results [6].

## 5. Conclusions

In summary, we provide evidence that ritonavir-induced activation of RB is linked to inhibition of the AKT pathway by a common signaling mechanism, resulting in down-regulation of S phase genes. This may prove useful in the chemotherapeutic management of PDAC in conjunction with other agents.
